# Increased standardised incidence ratio of cardiovascular diseases among colorectal cancer patients

**DOI:** 10.1007/s00384-022-04129-3

**Published:** 2022-03-17

**Authors:** Hsin-Yin Hsu, Yih-Jong Chern, Cheng-Tzu Hsieh, Tzu-Lin Yeh, Ming-Chieh Tsai, Chia-Chun Wang, Bo-Yu Hsiao, Jing-Rong Jhuang, Chun-Ju Chiang, Wen-Chung Lee, Kuo-Liong Chien

**Affiliations:** 1 Department of Family Medicine, Taipei MacKay Memorial Hospital, Taipei, Taiwan; 2 Institute of Epidemiology and Preventive Medicine, College of Public Health, National Taiwan University, Taipei, Taiwan; 3 Department of Medicine, MacKay Medical College, New Taipei City, Taiwan; 4 Division of Colon and Rectal Surgery, Department of Surgery, Chang Gung Memorial Hospital, TaoYuan, Taiwan; 5 Department of Family Medicine, Hsinchu MacKay Memorial Hospital, Hsinchu, Taiwan; 6 Division of Endocrinology, Department of Internal Medicine, Tamsui Branch, MacKay Memorial Hospital, New Taipei City, Taiwan; 7 Division of Radiation Oncology, Department of Oncology, National Taiwan University Hospital, Taipei, Taiwan; 8 Taiwan Cancer Registry, Taipei, Taiwan; 9 Innovation and Policy Center for Population Health and Sustainable Environment, College of Public Health, National Taiwan University, Taipei, Taiwan; 10 Department of Internal Medicine, National Taiwan University Hospital, Taipei, Taiwan

**Keywords:** Colorectal cancer, Cardiovascular diseases, Cardio-oncology

## Abstract

**Purpose:**

Evidence regarding the relationship between colorectal cancer and the risk of cardiovascular disease (CVD) is limited. Thus, in this study, we aimed to determine the standardised incidence ratio (SIR) of CVDs in colorectal cancer patients in Taiwan.

**Methods:**

A population-based cohort study enrolling the incident colorectal cancer population based on the Cancer Registry Database from 2007 to 2016 was conducted (*n* = 94,233, mean age: 62.4 years, 43.0% women). New cases of CVD, including coronary heart disease and ischemic stroke, through 31 December 2018 were obtained from the National Health Insurance Research Database and National Death Registry. Compared with the general population (*n* = 1,977,659, mean age: 44.3 years, 49.6% women), age- and sex-specific SIRs for CVDs were calculated by the time since diagnosis.

**Results:**

A total of 6852 cardiovascular events occurred in colorectal cancer patients during a median follow-up of 4.4 years. The SIR of CVD was highest in the first year after diagnosis (SIR: 1.45, 95% confidence interval: 1.39–1.50); however, this decreased to the same value as that of the general population in later years. Similar patterns were observed for the SIR of coronary heart disease. However, the SIR of ischemic stroke among colorectal cancer patients was low from the second year following cancer diagnosis.

**Conclusions:**

Colorectal cancer patients are at an increased risk of developing CVD, especially coronary heart disease, during the first 3 years following colorectal cancer diagnosis.

**Supplementary information:**

The online version contains supplementary material available at 10.1007/s00384-022-04129-3.

## Introduction

Colorectal cancer is one of the most common types of cancer and is also the leading cause of cancer-related mortality in the world. The yearly incidence of colorectal cancer has been the highest among the leading types of cancer in Taiwan since 2007, with an age-standardised incidence of 42.9 per 100,000 people in 2017 [1, 2]. Furthermore, colorectal cancer is the third most common cause of cancer-related death in Taiwan, preceded only by lung and liver cancer. The common risk factors for colorectal cancer include older age, personal or family history of colorectal cancer, genetic changes that increase the risk of colorectal cancer (e.g. *HNPCC* and *APC*), chronic ulcerative colitis, unhealthy diet (high-fat, low-calcium, low-folic acid, and low-fibre diet, and lack of fruits and vegetables), smoking, and insufficient physical activity [3–5].

The treatment of colorectal cancer is closely related to the cancer stage. The staging of colorectal cancer is based on the size of the tumour, extent of tumour invasion, size and location of involved lymph nodes, and metastasis. The treatment for colorectal cancer is changing rapidly due to medical advances and includes such options as surgery; chemotherapy; radiation therapy; immunotherapy; and, recently, cell therapy [6–9].

Cardiovascular disease (CVD) is the leading cause of death globally [10, 11]. The traditional risk factors of CVD include hypertension, diabetes, hyperlipidaemia, family history of atherosclerotic CVD, old age, and smoking [12]. In addition to traditional risk factors, there is evidence demonstrating that colorectal cancer patients may be at higher risk for CVDs [13–16]. The association between colorectal cancer and an elevated cardiovascular risk may be explained by the fact that colorectal cancer and CVDs share common risk factors, including older age, obesity, a sedentary lifestyle, and smoking [17, 18]. Furthermore, tumour burden, as well as the side effects associated with colorectal cancer treatment, may cause subsequent cardiovascular stress [18–21]. Although previous studies have found that CVD may be more prevalent in patients with colorectal cancer than in the general population, the evidence is limited. Furthermore, only a few studies have examined the potential time-varying association between CVD and colorectal cancer following cancer diagnosis [14].

We conducted a retrospective cohort study to investigate whether colorectal cancer patients are at a higher risk of developing CVDs than the general population and to assess if the cardiovascular risk varies over time. Furthermore, we also aimed to examine potential factors, such as sex, stage, or treatment status, that affect the risk of CVDs among patients with colorectal cancer.

## Methods

### Study design and population

In this retrospective population-based cohort study, the study population included colorectal cancer patients registered in the long form of the Taiwan Cancer Registry database from 2007 to 2016. Exclusion criteria were age < 20 years or > 85 years, evidence of previous CVD, diagnosis of other cancers, and presence of missing data. The follow-up period was from the date of colorectal cancer diagnosis to the primary outcome date, date of death, or 31 December 2018, whichever occurred first.

This study was approved by the Institutional Review Board of Mackay Memorial Hospital (reference number: 20MMHIS479e) and was conducted in accordance with the Declaration of Helsinki. The Taiwan Cancer Registry data collection followed the Institutional Review Board regulation, and informed consent was obtained accordingly.

The data sources for this study were the Taiwanese National Health Insurance Research Database (NHIRD) of the Ministry of Health and Welfare and the Taiwan Cancer Registry Centre. The data were based on the long form of the Taiwan Cancer Registry database from 2007 to 2016 and the general population database (randomly sampled from 2007 Taiwan Household Data), both of which are linked to the NHIRD [22, 23]. The Taiwan Cancer Registry database is a high-quality nationwide incident cancer registry system with 98.4% completeness, 93.0% morphologically verified cases, 45.1% mortality versus incidence ratio, 0.9% death certificate-only case data, and 14-month data timelines [22, 24]. The long form database of the Taiwan Cancer Registry collects cancer-related information, including cancer stage, the first course of treatment, and detailed clinical data related to patient care [22]. The NHIRD contains data on the utilisation of all NHI resources, including outpatient visits, hospital care, and prescribed medications.

### Definition of exposure

Colorectal cancer was identified based on the International Classification of Diseases for Oncology diagnosis codes, including C18 for colon cancer, C19 for rectosigmoid junction cancer, and C20 for rectal cancer (Table S1) [25].

### Definition of outcomes

Each participant in the long form of the Taiwan Cancer Registry database was linked to the NHIRD and National Death Registry to determine their outcomes. The primary outcome was the occurrence of the first major cardiovascular event, including hospitalisation for coronary heart disease, an arterial revascularisation procedure, hospitalisation for ischemic stroke, or confirmed death from coronary heart disease or ischemic stroke (Table S2).

### Statistical analyses

Baseline characteristics of colorectal cancer patients and the general population are presented as categorical (*n* (%)) and continuous variables (mean ± SD) according to sex. *t*-test or chi-square test was used to assess differences in baseline characteristics between the groups.

The observed number of CVDs among the cohort was compared with the expected number of CVDs derived from the incidence rate among the general population aged between 20 and 85 years in Taiwan during each year since cancer diagnosis. Age- (10-year strata), sex-, and year since cancer diagnosis-specific standardised incidence ratios (SIRs) with 95% confidence intervals (CIs) were obtained by calculating the ratio of observed cases to expected cases and applied to the study cohort’s experience of person-years of follow-up in each specific stratum and then summed across different strata [26, 27]. Subgroup analyses stratified by sex, staging, and treatment status were performed to explore potential populations with a more prominent risk of CVDs.

The level of statistical significance was set at a two-tailed alpha level < 0.05. Analyses were performed using SAS version 9.4 (SAS Institute, Cary, NC, USA).

## Results

A total of 121,801 colorectal cancer patients diagnosed between 2007 and 2016 were enrolled in this study. A total of 27,568 patients were excluded, including 7762 who were younger than 20 or older than 85 years and 19,806 who had established CVD prior to colorectal cancer diagnosis, or missing data. The mean age of the colorectal cancer patient population was 62.4 (± 12.4) years, and 43.0% were women. Using the same exclusion criteria, 1,977,659 participants in the general population were enrolled (mean age 44.3 years, 49.6% women). The prevalence of hypertension, diabetes, and hyperlipidaemia in the colorectal cancer patient population was 51.6%, 20.5%, and 25.4%, respectively, which were all higher than those in the general Taiwanese population. Table 1 shows the basic characteristics of the colorectal cancer patients.

**Table 1:** Distribution of baseline demographic, clinical characteristics in the study population

	Colorectal cancer population *n* = 94,233	General population *n* = 1,977,659
Women (%)	40,522 (43.0)	980,941 (49.6)
Age (years)		
20–39	4162 (4.4)	867,743 (43.9)
40–64	47,881 (50.8)	863,608 (43.7)
≥65	42,190 (44.8)	246,308 (12.5)
Staging (%)		
0	14,181 (15.4)	
I	15,868 (17.2)	
II	18,252 (19.8)	
III	25,314 (27.5)	
IV	18,593 (20.2)	
The first course of treatment*		
Surgery (%)	84,588 (89.8)	
Chemotherapy (%)	51,091 (54.2)	
Radiotherapy (%)	12,063 (12.8)	
Hypertension (%)	48,578 (51.6)	
Diabetes Mellitus (%)	19,308 (20.5)	
Hyperlipidemia (%)	23,924 (25.4)	

^*^The first course of treatment for colorectal cancer extracted from the Taiwan Cancer Registry defined as all documented treatments performed during the initial treatment plan and the maintenance therapy prior to disease progression or recurrence

During a median follow-up of 4.4 years, incident cardiovascular events (*n* = 6852), coronary heart disease (*n* = 4684), and ischemic stroke (*n* = 3415) occurred in the colorectal cancer patient population. Compared with the Taiwanese general population, the overall cardiovascular risk was slightly but significantly lower among colorectal cancer patients, with an SIR of 0.92 (95% CI, 0.90–0.94). The overall ischemic stroke risk was also significantly lower than that of the general population, and the SIR was 0.81 (95% CI: 0.78–0.84). The overall coronary heart disease risk among colorectal cancer patients was similar to that in the general population (SIR 1.01, 95% CI: 0.98–1.03).

**Fig. 1:**
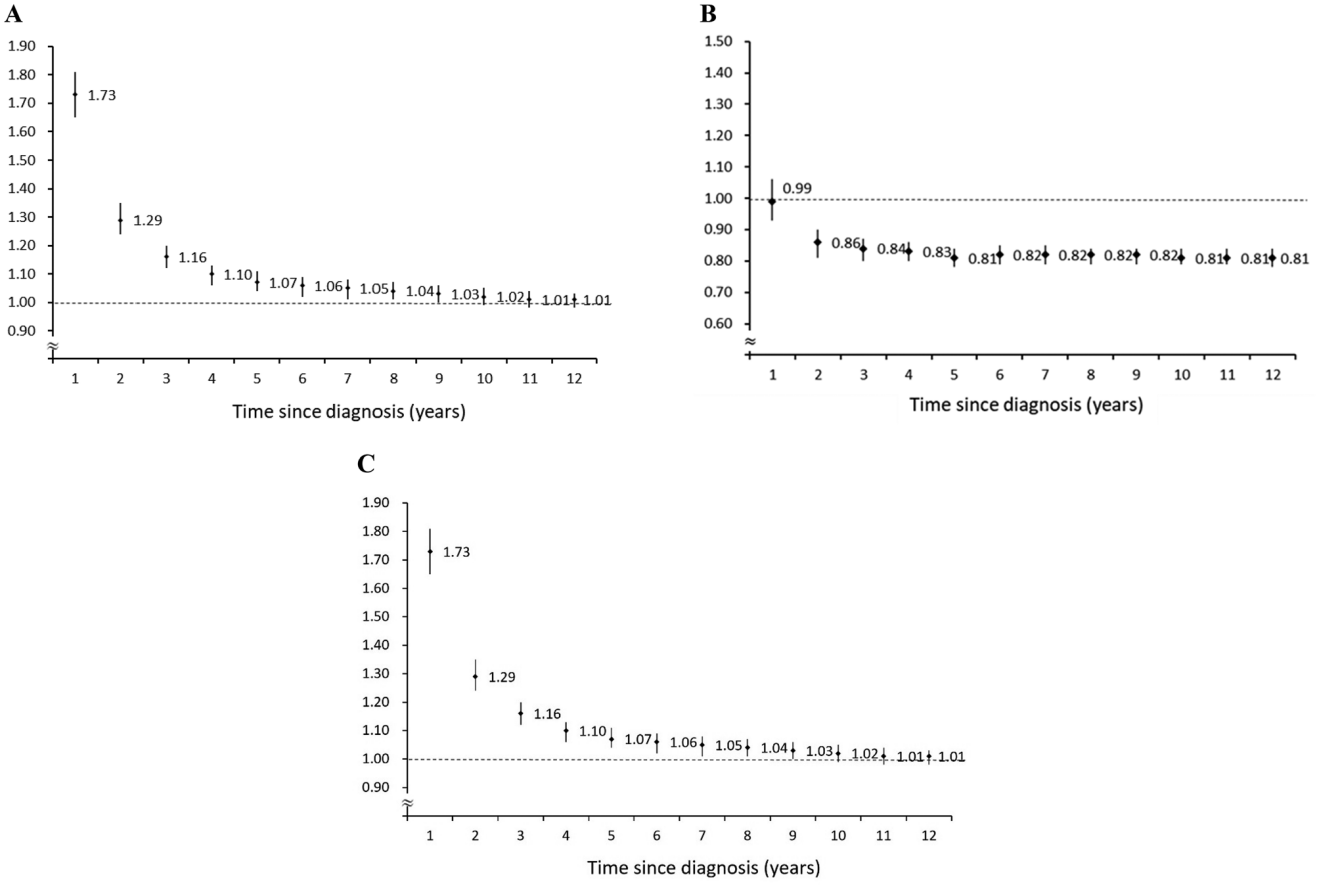
Standardised incidence ratios (SIRs) for cardiovascular diseases among colorectal cancer patients based on time since diagnosis. **A** SIRs of coronary heart disease in colorectal cancer patients. **B** SIRs of ischemic stroke in colorectal cancer patients. **C** SIRs of cardiovascular disease in colorectal cancer patients

We next considered the risk of CVD based on the time since colorectal cancer was diagnosed. The SIR of CVD within the first 3 years after diagnosis of colorectal cancer was significantly higher than that in the general Taiwanese population. The SIR was highest in the first year after colorectal cancer diagnosis (SIR 1.45, 95% CI, 1.39–1.50) and declined annually to a lower risk of cardiovascular risk than that of the general Taiwanese population (Fig. 1A). The risk of coronary heart disease significantly increased within the first 9 years after colorectal cancer was diagnosed (Fig. 1B). However, the coronary heart disease risk was highest within the first year after colorectal cancer diagnosis (SIR 1.73, 95% CI, 1.65–1.81) and regressed to a value similar to that of the general population. As shown in Fig. 1C, colorectal cancer patients had an ischemic stroke risk similar to that of the Taiwanese population in the first year after cancer diagnosis (SIR 0.99, 95% CI, 0.93–1.06) but had a lower ischemic stroke risk thereafter.

**Fig. 2:**
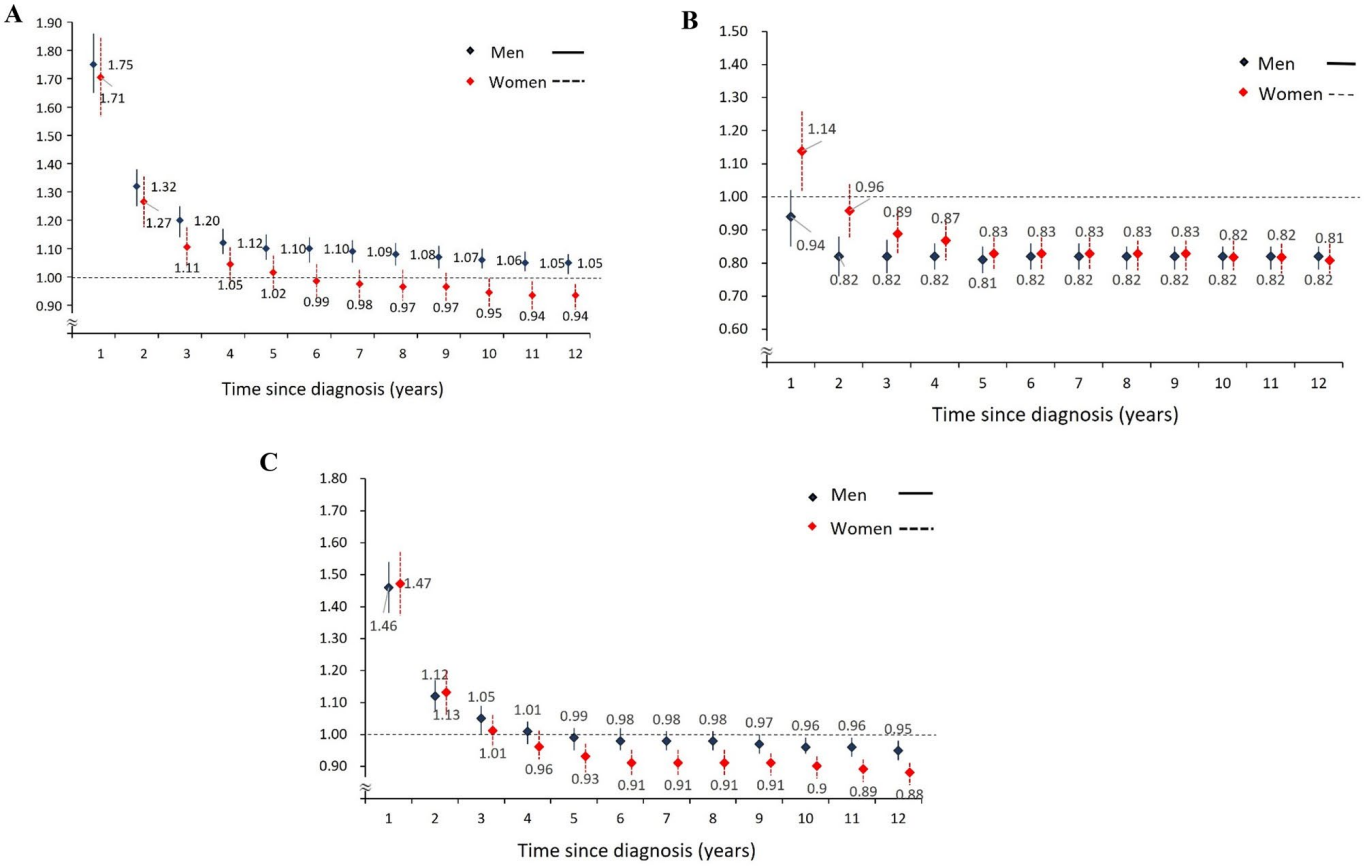
Standardised incidence ratios (SIRs) for cardiovascular diseases among colorectal cancer patients based on time since diagnosis. **A** SIRs of coronary heart disease in colorectal cancer patients. **B** SIRs of ischemic stroke in colorectal cancer patients. **C** SIRs of cardiovascular disease in colorectal cancer patients, stratified by sex

The SIRs of CVD were consistent between men and women. Similar patterns were noted in the SIRs of patients with ischemic stroke. However, a higher risk of coronary heart disease was found in men than in women (Fig. 2A–C).

**Fig. 3:**
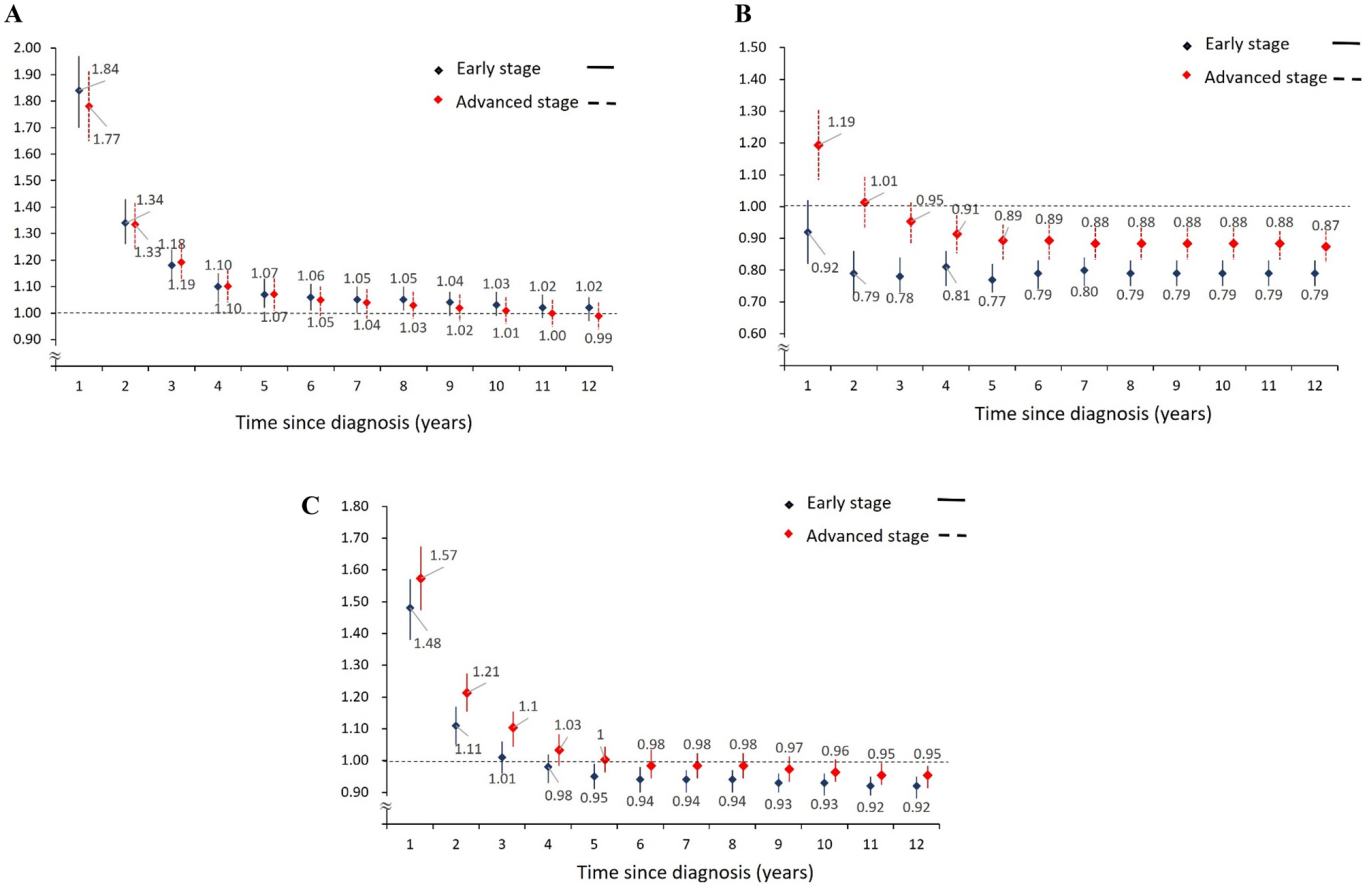
Standardised incidence ratios (SIRs) for cardiovascular diseases among colorectal cancer patients based on time since diagnosis. **A** SIRs of coronary heart disease in colorectal cancer patients. **B** SIRs of ischemic stroke in colorectal cancer patients. **C** SIRs of cardiovascular disease in colorectal cancer patients, stratified by stage

In the subgroup analysis stratified by colorectal cancer stage, more prominent cardiovascular risk was noted among patients with advanced colorectal cancer within the first 3 years after colorectal cancer diagnosis (Fig. 3A). As shown in Fig. 3B, a similar risk pattern was noted for ischemic stroke. However, the SIRs of coronary heart disease were consistent between patients with advanced- and early-stage colorectal cancer (Fig. 3C). We also performed subgroup analysis stratified by treatment status. A higher coronary heart disease risk is noted in the patients not receiving chemotherapy, radiotherapy, or surgery which was noted since the 2nd year following the cancer diagnosis. However, no significant difference in the risk of coronary heart disease was observed between patients with different treatment statuses around the time of cancer diagnosis (Fig. S1). The results of the subgroup analysis stratified by treatment status in ischemic stroke showed similar patterns with the results in coronary heart disease.

## Discussion

This study suggests a positive association between colorectal cancer and the incidence of coronary heart disease, especially within the first 3 years after colorectal cancer diagnosis. However, a significantly lower risk of ischemic stroke was noted among colorectal cancer patients. Compared with the general population, the risk of cardiovascular morbidity or mortality from either coronary heart disease or ischemic stroke among colorectal cancer patients was higher in the first 3 years after colorectal cancer diagnosis, especially in those with more advanced cancer.

Previous studies have demonstrated that cancer patients have a higher risk of CVD [18, 28, 29]. However, limited evidence has specifically elucidated the relationship between colorectal cancer and cardiovascular risk [13, 15]. In a cohort study including 1749 long-term colorectal cancer survivors and 6480 healthy individuals, colorectal cancer survivors had a two to four times higher risk of CVDs after a follow-up of 10 years, especially older patients with more comorbidities (hypertension, hazard ratio [HR] 2.87, 95% CI 2.59–3.11; heart disease HR 2.66, 95% CI 2.37–2.98; cerebrovascular disease, HR 2.98, 95% CI 2.36–3.76) [13]. In 2015, a South Korean cross-sectional study found that colorectal tumours were significantly correlated with an increase in the cardiac calcification index (odds ratio, 1.66; 95% CI, 1.05–2.64) [30]. Another retrospective cohort study on 65,829 individuals from the health insurance database of the USA from 2001 to 2017 classified into colorectal cancer and non-cancer groups matched by age, sex, ethnicity, and multiple comorbidities showed that the cumulative incidence of stroke in the colorectal cancer group was 3.3% (95% CI 3.2–3.4), while it was 1.3% (95% CI 1.2–1.4) in the non-cancer group [31].

In the present study, there was no significant difference in overall cardiovascular risk among patients with colorectal cancer. However, the novelty of this study is that it explored the time-related association between colorectal cancer and cardiovascular risk. The highest risk of CVD in the colorectal cancer population was found within the first year after colorectal cancer was diagnosed, and the risk gradually decreased over time to nearly the same risk level as that in the general population. The risk of coronary heart disease in colorectal cancer patients remained significantly higher than that in the general populations within 10 years after cancer diagnosis. However, the ischemic stroke risk was lower among colorectal cancer patients beginning the second year after cancer diagnosis. Previous studies have revealed no difference or only a mildly increased frequency of ischemic strokes around the time of cancer diagnosis in cancer populations compared with that in non-cancer populations [31–33]. The discrepancy between our results and those of previous studies may be explained by the fact that the original short-term high ischemic stroke risk in colorectal cancer patients is offset or even reversed by intensive integrated medical care. On the other hand, the effect of intensive care on the risk of coronary heart disease was not so obvious as that on ischemic stroke which may have resulted from colorectal cancer patients having a much higher risk from coronary heart disease than ischemic stroke [14].

The association between colorectal cancer and the increased risk of CVDs may be explained by at least four potential mechanisms. First, colorectal cancer and CVD share common risk factors, such as older age, obesity, sedentary lifestyle, and smoking [17, 18]. Second, the hypercoagulable state associated with the tumour burden in colorectal cancer may increase the risk of thromboembolic events [18, 34]. Third, multiple treatments, including surgery, chemotherapy, and radiotherapy, around the time of cancer diagnosis, may pose a higher risk of cardiovascular toxicity to patients with higher cardiovascular risk [19, 35–37]. Fourth, colorectal cancer, as well as the accompanying treatments, may worsen the risk of CVD in patients [19, 21]. Our study showed a more prominent CVD risk in advanced-stage patients and in the patients not receiving chemotherapy, radiotherapy, or surgery since the 2nd year following the cancer diagnosis. The risk discrepancy was much more obvious in different surgery statuses. According to Cancer Registry Data report, about 90% of colorectal cancer patients received surgical treatment; those not receiving surgical treatment are more likely to be end-stage patients with more comorbidities which may pose a higher cardiovascular risk [2]. However, treatment around the time of cancer diagnosis probably reduces the risk discrepancy between different treatment statuses. Although the subgroup results implied that higher tumour burden status may contribute more to cardiovascular risk in colorectal cancer patients, further study designs with more detailed treatment information or statistical methods may be warranted to explore the factors causing increased cardiovascular risk in colorectal cancer patients.

Integrated medical care may be needed for colorectal cancer patients. Screening and management of cardiovascular risk factors in colorectal cancer patients cannot be ignored [13, 38, 39]. For these patients, the periods of treatment and post-treatment surveillance are usually long, and they need regular follow-up at clinics to monitor for cancer recurrence. Therefore, primary care providers of colorectal cancer patients should be aware of the patient’s symptoms and signs of CVD and refer the patient to the appropriate specialist if CVD is suspected. In addition, chemotherapy that is associated with higher risk of cardiotoxicity should be administered with caution, especially in patients with higher CVD risk, such as elderly patients and patients with diabetes, hypertension, or hyperlipidaemia [13, 35, 39–41]. Primary care providers should strongly consider continuous monitoring and potential chemoprophylaxis to prevent the development of CVDs in colorectal cancer patients, especially in the first 3 years following cancer diagnosis [39, 42].

The present study had several strengths. First, this is the first extensive investigation of colorectal cancer and CVD risk, including coronary heart disease and ischemic stroke, in an Asian population. Previous studies have provided evidence of an association between colorectal cancer and a potentially increased cardiovascular risk in Asian populations, but no study has explored the time-related relationship between colorectal cancer diagnosis and the risk of CVD. Second, the Taiwan Cancer Registry Database is a high-quality database, and the general population was randomly selected from the entire Taiwanese population, which made both the colorectal cancer and general populations in the present study nationally representative [24]. CVD outcomes were ascertained based on the NHIRD database. Therefore, this study provides robust evidence that colorectal cancer is positively correlated with CVD risk.

However, some limitations must be noted. The cohort study only included colorectal cancer patients and the general population in Taiwan, leading to a lack of external generalisability, because cardiovascular risk might vary according to race, age, or cancer treatment strategy. In addition, information regarding comorbidities in the general population were not available; thus, we applied age- and sex-specific SIRs to present the association between colorectal cancer and CVD risk. This may lead to residual confounding since certain important covariates, such as comorbidities or tumour burden, were not considered. However, we conducted subgroup analyses to ameliorate potential confounding biases. Moreover, the competing risk of death in the colorectal cancer population was present; however, ignoring the competing risk may bias the true effect between colorectal cancer and cardiovascular risk. Finally, further study designs with more detailed treatment information or statistical methods are required to examine the factors contributing to increased cardiovascular risk in colorectal cancer patients.

## Conclusions

This cohort study demonstrated no significant difference in overall cardiovascular risk among patients with colorectal cancer. However, the risk of CVD associated with colorectal cancer seemed to vary based on time since diagnosis. Compared with the general population, colorectal cancer patients have a higher risk of developing CVD, especially coronary heart disease, in the first 3 years after colorectal cancer diagnosis. The CVD risk was more prominent in patients with advanced-stage disease.

## Supplementary Information

Below is the link to the electronic supplementary material.

Supplementary file1 (DOCX 444 KB)

## Data Availability

The data generated and/or analysed during the current study are not publicly available due to the terms of consent to which the participants agreed but are available from the authors upon reasonable request and with permission from the Health Promotion Administration at the Ministry of Health and Welfare in Taiwan.

## Declarations

### Disclaimer

The funders had no role in the study design, data collection and analysis, decision to publish, or preparation of the manuscript. The content of this research may not represent the opinion of the Health Promotion Administration, Ministry of Health and Welfare in Taiwan.
