# Supplementary material for: Increased standardised incidence ratio of cardiovascular diseases among colorectal cancer patients
Source: Int J Colorectal Dis. 2022 Mar 17;37(4):887–94. doi: 10.1007/s00384-022-04129-3 (PMC8976771; doi:10.1007/s00384-022-04129-3)
Supplement: Supplementary file 1 — Supplementary file1 (DOCX 444 KB) [file 384_2022_4129_MOESM1_ESM.docx]

Table S1 Definitions of the clinical outcomes in the study cohort

| Outcome | Definitions | ICD-9 codes | ICD-10 codes | Procedure codes |
| --- | --- | --- | --- | --- |
| Coronary heart disease | ≥ 1 discharge diagnosis of ICD-9 or 10 codes or procedure codes | 410, 411, 414.00, 414.01, 414.02, 414.03, 414.04, 414.05,  v45.81, v45.82 | I21.01, I21.02, I21.09, I21.11, I21.19, I21.29, I21.21, I21.4, I21.3, I22.0, I22.1, I22.2, I22.8, I22.9, I20.0, I24.0, I24.1, I24.8, I24.9, I25.10, I25.750, I25.751, I25.758, I25.759, I25. 811, I25.10, I25.110, I25.111, I25.118, I25.119, I25.750, I25.751, I25.758, I25.759, I25.760, I25.761, I25.768, I25.769, I25. 811, I25.710, I25.711, I25.718, I25.719, I25.812, I25.730, I25.731, I25.738, I25.739, I25.720, I25.721, I25.728, I25.729, I25.700, I25.701, I25.708, I25.709, I25.730, I25.731, I25.738, I25.739, I25.760, I25.761, I25.768, I25.769, I25.790, I25.791, I25.798, I25.799, I25.810, I25.812, Z95.1, Z95.5, Z98.61 | 33076B, 33077B, 33078B, 68023B, 68024B, 68025B, N26002, N26003 |
| Ischemia stroke | Discharge diagnosis of the ICD-9 or ICD-10 codes | 433, 434, 435, 436, 437.1, 437.9 | 165.1,,I63.02, I63.12, I65.21, I63.22, I65.1, I65.23, I65.29 , I63.031, I63.032, I63.039, I63.131, I63.132, I63.139, I63.231, I63.232, I63.239, I65.01, I65.02, I65.03, I65.09, I63.011, I63.012, I63.019, I65.22, I63.111, I63.112, I63.119, I63.211, I63.212, I63.219, I65.8, 163.09, I63.19, I63.59, I65.9, I63.00, 163.10, I63.20, I63.29, I66.01, I66.02, I66.03, I66.09, I66.11, I66.12, I66.13, I66.19, I66.21, I66.22, I66.23, I66.29, I66.3, I63.30, I63.311, I63.312, I63.319, I63.321, I63.322, I63.329, I63.331, I63.332, I63.339, I63.341, I63.342, I63.349, I63.39, I63.6, I66.01, I66.02, I66.03, I66.09, I66.11, I66.12, I66.13, I66.19, I66.21, I66.22, I66.23, I66.29, I66.3, I66.9, I66.40, I66.411, I66.412, I66.419, I66.421, I66.422, I66.429, I66.431, I66.432, I66.439, I66.441, I66.442, I66.449, I66.49, I66.01, I66.02, I66.03, I66.09, I66.11, I66.12, I66.13, I66.19, I66.21, I66.22, I66.23, I66.29, I66.3, I66.8, I66.9, I63.50, I63.511, I63.512, I63.519, I63.521, I63.522, I63.529, I63.531, I63.532, I63.539, I63.541, I63.542, I63.549, I63.59, I63.8, I63.9, G45.0,G45.8,G45.1,G45.2, G46.0, G46.1, G46.2, G45.9, I67.841, I67.848, I67.89, I67.81, I67.82, I67.89, I67.9 |  |
| Cardiovascular disease | Coronary heart disease or ischemic stroke morbidity or mortality |  |  |  |

ICD-9: International Classification of Diseases-9^th^ revision; ICD-10: International Classification of Diseases-10^th^ revision

Table S2 Definitions of the colorectal cancer population the study cohort

| Tumor site | ICD-O-3 | Subsite |
| --- | --- | --- |
| Colon | C18.0 | Cecum |
|  | C18.1 | Appendix |
|  | C18.2 | Ascending colon |
|  | C18.3 | Hepatic flexure of colon |
|  | C18.4 | Transverse colon |
|  | C18.5 | Splenic flexure of colon |
|  | C18.6 | Descending colon |
|  | C18.7 | Sigmoid colon |
|  | C18.8 | Overlapping lesion of colon |
|  | C18.9 | Colon, NOS |
| Rectosigmoid junction | C19.9 | Rectosigmoid junction |
| Rectum | C20.9 | Rectum, NOS |

Figure S1 Standardised incidence ratios (SIRs) for coronary heart disease among colorectal cancer patients based on time since diagnosis, stratified by (A) chemotherapy treatment status (B) radiotherapy treatment status (C) surgery treatment status.


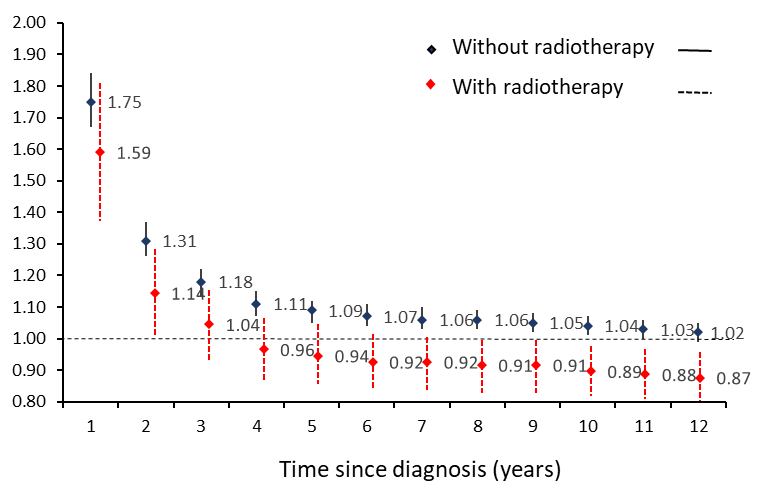

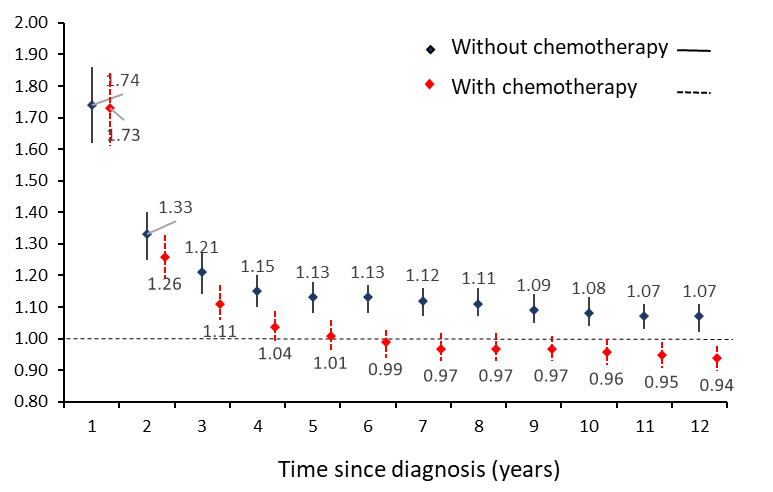

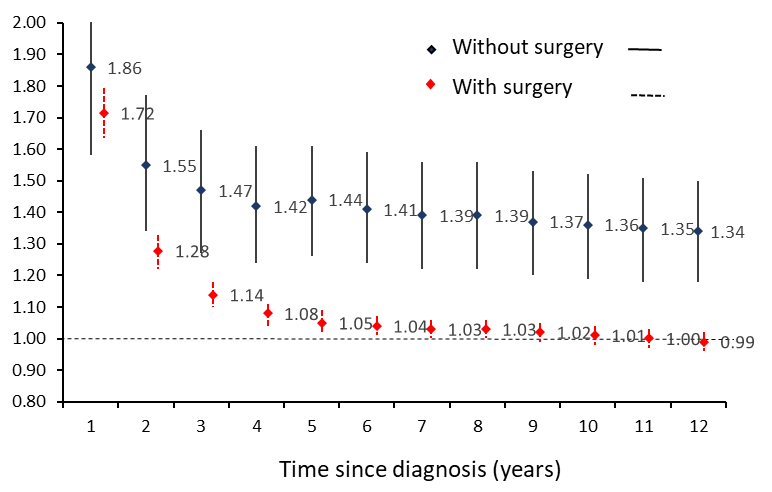


**Figure S1A**

**Figure S1B**

**Figure S1C**
